# Schwannome bénin du nerf grand sciatique, à propos de 2 cas

**DOI:** 10.11604/pamj.2014.18.252.4684

**Published:** 2014-07-26

**Authors:** Mohammed Chahbouni, Issam Eloukili, Mohamed Ali Berrady, Moulay Omar Lamrani, Mohamed Kharmaz, Farid Ismail, Mustapha Mahfoud, Ahmed El Bardouni, Mohamed Saleh Berrada, Mouradh El Yaacoubi

**Affiliations:** 1 Service de Traumatologie-Orthopédie, CHU Ibn Sina, Rabat, Maroc

**Keywords:** Tumeur, Cellule de schwan, IRM, Chirurgie, Tumor, Cell Schwan, MRI, Surgery

## Abstract

Les tumeurs primitives des nerfs périphériques représentent 1 à 2% des tumeurs des tissus mous. Les schwannomes sont en règle des tumeurs isolées de taille modérée et de croissance lente. Il convient de distinguer le schwannomes bénin et le neurofibrome des tumeurs malignes survenant généralement au cours d'une maladie de Recklinghausen. L'IRM permet d'orienter le diagnostic en mettant en évidence une tumeur de même signal que le tissu musculaire. Le traitement idéal de ces tumeurs consiste en une énucléation chirurgicale avec dissection soigneuse des faisceaux nerveux avoisinants. Nous rapportons deux cas d'un schwannome bénin développé aux dépens du nerf grand sciatique.

## Introduction

Les tumeurs primitives des nerfs périphériques représentent 1 à 2% des tumeurs des tissus mous. Il convient de distinguer le schwannomes bénin et le neurofibrome des tumeurs malignes survenant généralement au cours d'une maladie de Recklinghausen. Nous rapportons deux cas d'un schwannome bénin développé aux dépens du nerf grand sciatique. L'origine nerveuse de la tumeur ayant été suspectée en préopératoire sur ses caractéristiques cliniques et précisée aux données de l'imagerie par résonance magnétique (IRM).

## Patient et observation

### Observation 1

**Figure 1:**
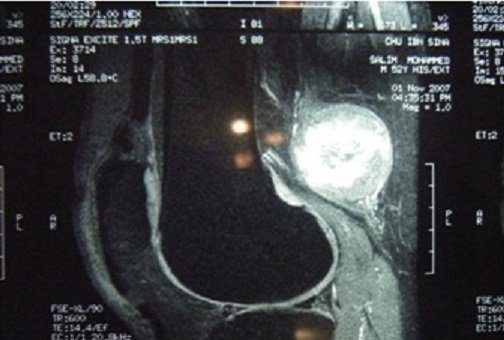
Coupe sagittale de IRM montrant le processus tumoral au dépend du nerf sciatique en séquence ponderée T2

**Figure 2:**
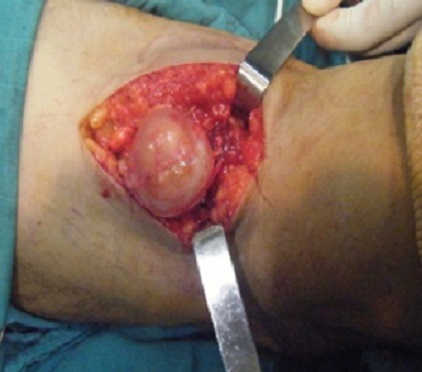
Vue opératoire du schwannome du nerf sciatique

Patient de 48 ans, sans ATCD pathologiques notables, qui présentait depuis 2 ans une masse du creux poplité, rétro condylienne, gauche se manifestant par des paresthésies et des décharges électriques irradiant vers la jambe. Cliniquement il s'agit d'une masse dure de 4 cm à peu près, adhérente au plan profond, la mobilité du genou est complète. La radiographie standard est normale. Une échographie a été réalisée qui a évoqué une tumeur aux dépens du grand sciatique. Le bilan radiologique a été complété par une IRM qui a bien visualisé la tumeur (Figure 1). Le patient a bénéficié d'un traitement chirurgical basé sur l'exérèse de la tumeur (Figure 2), la pièce opératoire a été envoyée pour une étude anatomo-pathologique dont le résultat était en faveur d'un schwannome bénin. L’évolution était favorable après un recul de 8 mois.

### Observation 2

**Figure 3:**
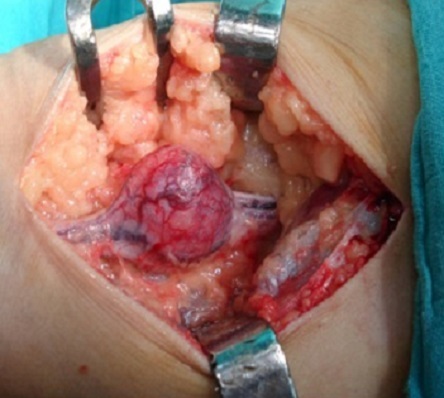
Vue opératoire du schwannome du nerf sciatique

Patiente de 53 ans, sans ATCD pathologiques notables, présentant depuis 3 ans une masse du creux poplité droit augmentant progressivement de taille avec des paresthésies et des décharges électriques irradiant vers la jambe. Cliniquement, il s'agit d'une masse dure de 5 cm de diamètre, fixe par rapport au plan profond. La radiographie standard était normale, le bilan a été complété par une IRM qui a mis en évidence une tumeur aux dépens du nerf sciatique. Un traitement chirurgical basé sur l'exérèse de la tumeur a été réalisé. La pièce opératoire (Figure 3) a été envoyée pour étude anatomo-pathologique qui a confirmé le diagnostic d'un schwannome bénin.

## Discussion

Les schwannomes bénins; autrefois appelés neurinomes, sont les plus fréquentes des tumeurs nerveuses [1]. Ils se développent au dépens des cellules de schwann, formant une prolifération macroscopiquement lisse, arrondie, jaunâtre et encapsulée [1, 2]. Ils sont facilement clivables des faisceaux nerveux qu'ils refoulent anas les envahir; permettant ainsi une énucléation complète de la tumeur [1, 2]. La transformation maligne est exceptionnelle voire discutée, elle surviendrait principalement dans le cadre d'une maladie de Recklinghausen [1–3]. Ces tumeurs surviennent avec prédilection chez l'adulte de 20 à 50 ans, et toujours indifféremment l'homme et la femme. Elles de localisent préférentiellement à la face antérieure des membres supérieurs, classiquement au niveau des grands troncs nerveux [2–4]. Les membres inférieurs ne sont que rarement le siège de schwannomes bénins et posent plus le problème diagnostic [4–6], ils intéressent dans cette localisation aussi bien les grands troncs nerveux que les nerfs sensitifs superficiels. Il faut savoir évoquer le diagnostic devant une douleur ou des paresthésies d'un membre inférieur sans anomalie clinique évidente.

Les schwannomes sont en règle des tumeurs isolées de taille modérée et de croissance lente, palpables lorsqu'ils sont volumineux ou superficiels. Le délai d'apparition des premiers signes est généralement long; souvent plusieurs années [7, 8]. Les douleurs à type de paresthésie sont souvent les premières et uniques manifestations [7, 8] comme le cas de nos patients. Les déficits sensitifs et moteurs objectifs sont rares en raison du caractère non infiltrant de la tumeur, ce qui explique que l'exploration électromyographique soit généralement normale. L'IRM permet d'orienter le diagnostic en mettant en évidence une tumeur de même signal que le tissu musculaire sur les séquences pondérées en T1 et de signal très intense en T2 avec quelques plages centrales d'hyposignal, mais elle ne permet pas de différencier les schwannomes des neurofibromes [7–9], c'est l'histologie qui permet de confirmer le diagnostic. Histologiquement, le neurofibrome solitaire représente le principal diagnostic différentiel.

Le traitement idéal de ces tumeurs consiste en une énucléation chirurgicale avec dissection soigneuse des faisceaux nerveux avoisinants [8–10] comme ce fût le cas de nos patients; cependant la simple résection de la tumeur avec son nerf d'origine est parfois possible en cas de localisation distale sur un nerf sensitif superficiel [8–10]. L’évolution est généralement favorable après résection chirurgicale.

## Conclusion

La localisation du schwannome bénin est rare au niveau du membre inférieur, et pose souvent des problèmes diagnostiques, cependant, les nouvelles investigations en imagerie et l’étude histologique, rendent le diagnostic facile, et la résection de la tumeur permet d'apporter des résultats spectaculaires.
