# Corrigendum: Schwannome bénin du nerf grand sciatique, à propos de 2 cas

**DOI:** 10.11604/pamj.2018.30.165.15870

**Published:** 2018-06-25

**Authors:** Mohammed Chahbouni, Issam Eloukili, Mohamed Ali Berrady, Moulay Omar Lamrani, Mohamed Kharmaz, Farid Ismail, Mustapha Mahfoud, Ahmed El Bardouni, Mohamed Saleh Berrada, Mouradh El Yaacoubi

**Affiliations:** 1 Service de Traumatologie-Orthopédie, CHU Ibn Sina, Faculté de Médecine et de Pharmacie de Rabat, Université Mohamed V, Rabat, Maroc

**Keywords:** Corrigendum, tumeur, cellule de Schwan, IRM, chirurgie

## Abstract

This Corrigendum corrects “Schwannome bénin du nerf grand sciatique, à propos de 2 cas” The Pan African Medical Journal. 2014;18:252. doi:10.11604/pamj.2014.18.252.4684.

## Corrigendum

The author affiliation in the published article [1] “Service de Traumatologie-Orthopédie, CHU Ibn Sina, Rabat, Maroc” is incomplete. It should read: “Service de Traumatologie-Orthopédie, CHU Ibn Sina, Faculté de Médecine et de Pharmacie de Rabat, Université Mohamed V, Rabat, Maroc”.
